# From physiological workload to motivation during a prolonged trail run: the mediating role of affective valence and arousal

**DOI:** 10.3389/fspor.2026.1805336

**Published:** 2026-07-01

**Authors:** Mélissa Muzeau, Andrew Flood, Jocelyn Mara, Nicholas Tam, Walter Staiano, Ben Rattray

**Affiliations:** 1 Reserach Institute of Sport and Exercise, University of Canberra, Canberra, ACT, Australia; 2 Discipline of Psychology, University of Canberra, Canberra, ACT, Australia; 3 Grounded Data Science, Canberra, ACT, Australia; 4 Sport Science, On AG, Zurich, Switzerland; 5 Department of Human Biology, University of Cape Town, Cape Town, South Africa; 6 Department of Physical Education and Sport, University of Valencia, Valencia, Spain; 7 Department of Psychology, University of Southern Denmark, Odense, Denmark; 8 Discipline of Sport and Exercise Science, University of Canberra, Canberra, ACT, Australia

**Keywords:** affective valence, endurance performance, fatigue, mediation analysis, motivation, psychology

## Abstract

**Intro:**

Motivation is critical for sustaining effort during prolonged exercise, yet the mechanisms underlying in-task fluctuations remain unclear. Affective responses may contribute to these fluctuations given their links with exercise intensity and motivation. This exploratory study investigated the relationship between physiological workload relative to speed and motivation, and the mediating role of affective responses.

**Methods:**

Fourteen trained male trail-runners completed two 90 min runs on a 2 km loop while maintaining an RPE of 14 on Borg's 6–20 scale. Heart rate and running speed were continuously recorded, and RPE, affective responses (valence and arousal), and motivation were assessed each lap. Physiological workload was indexed via the HR:Speed ratio. Bayesian mediation models quantified direct and indirect effects of physiological workload on motivation through valence and arousal.

**Results:**

A 1-unit increase in HR:Speed from the mean was associated with a 0.19-unit decrease in motivation. Higher physiological load was associated with 3.2 greater odds of lower valence, while evidence for changes in arousal was weaker. Higher valence and arousal tended to be associated with greater motivation. Mediation analysis suggested that 32% of the association between HR:Speed and motivation operated through affective responses with valence contributing most of the indirect effect. This indirect pathway appeared workload-dependant, with higher valence associated with greater motivation at lower intensity, but lower valence associated with reduced motivation at higher intensity.

**Conclusion:**

These finding provide preliminary evidence for a potential role of affective valence in motivational regulation during prolonged run. Further research is needed to clarify the robustness of these relationships and to determine whether intervention targeting affective responses may influence performance

## Highlights

- Rising physiological workload relative to speed seems to progressively reduce motivation in a prolonged trail run.
- Affective valence appears to partially account for the workload–motivation relationship, supporting motivation at low and undermining it at high intensity.
- Supporting positive affect and optimal arousal level may help athletes preserve motivation under increasing fatigue.

## 1 Introduction

The essence of competitive running is to cover a given distance in the shortest possible time. Achieving this outcome depends not only on physiological capacity but also on the athlete's willingness to exert and sustain effort. This willingness, often conceptualized as potential motivation, reflects the maximum effort an individual is ready to invest in a task (1–3). According to the motivation intensity theory, greater potential motivation enables greater effort investment, particularly when task outcomes are valued and success is perceived as attainable (4). Although motivation is often treated as a stable characteristic, evidence shows that it fluctuates dynamically during exercise and responds to changes in both internal states and environmental cues (5–7). However, the mechanisms underlying these fluctuations remain poorly understood.

The psychobiological model of endurance performance proposes that exercise intensity must be regulated when the perceived cost of effort outweighs potential motivation (65). This perceived cost may progressively increase as fatigue develops. This concept aligns with durability, or physiological resilience, which has recently been proposed as a fourth physiological determinant of endurance performance and is defined as the ability to resist fatigue and maintain performance over time (8–10). Reduced durability during prolonged exercise is characterised by an increasing physiological cost for a given external workload. In running, this phenomenon can be observed as a progressive decoupling between internal and external workload and quantified using the HR:speed ratio, reflecting the increase in heart rate required to maintain a given running speed. The HR:speed ratio has previously been used in trail-running contexts (11) and will be referred as a proxy of physiological workload in the present study.

Several theoretical frameworks suggest that endurance performance emerges from a continuous interaction between physiological workload, motivational resources and affective responses (12–14). Affect has been conceptualised as the most elementary consciously accessible feeling which, in interaction with motivation, regulates behaviour by orienting individuals toward beneficial stimuli and away from harmful ones (63). Affective responses can be characterised by the degree of pleasure or displeasure experienced (valence) and the level of perceived activation (arousal) (15, 16). Empirical evidence supports the predictions of these models. Affective valence has been shown to influence pacing strategies (17, 18), while more positive affective states have been associated with improved endurance performance (19, 20).

From a mechanistic perspective, affective responses appear to contribute to effort regulation through at least two complementary pathways. First, affective responses fluctuate systematically with exercise intensity. According to Dual–Mode Theory, as physiological workload increases, particularly beyond the glycolytic threshold, exercise is generally experienced as progressively more unpleasant due to the increasing influence of interoceptive physiological cues (21, 22). This shift toward negative valence is thought to act as a protective self-regulatory signal, indicating rising homeostatic disturbance and prompting athletes to reduce or terminate effort (20, 23, 24). Second, affect may not merely reflect physiological strain but shape the willingness to sustain effort (25). Recent evidence has suggested that affective valence may mediate the relationship between physiological load and the desire to reduce effort (26). However, this work has primarily been conducted in laboratory settings and has largely neglected the role of arousal, limiting both the ecological and theoretical scope of current understanding. This omission is particularly relevant given that arousal has been associated with task engagement (27), suggesting that it may play an important role in the regulatory process linking physiological workload and motivation. One theoretical framework particularly relevant to this question is Reversal Theory, which proposes that transient motivational states influence whether physiological arousal is interpreted as pleasant or unpleasant. According to this perspective, identical levels of arousal may be experienced as excitement in one motivational state and distress in another (28). Conversely, affective changes may themselves trigger reversals between motivational states, suggesting a dynamic and bidirectional relationship between affect and motivation during exercise.

The purpose of this exploratory study was to investigate how changes in physiological workload may relate to in-task motivation during a prolonged trail run and to explore whether affective responses may play a mediating role in this relationship. Grounded in affect-based models of effort regulation and motivational intensity theory, we tested the joint mediating roles of affective valence and arousal in an ecologically valid endurance context, with the aim to better understand how physiological strain, affective experience, and motivation may dynamically interact to regulate effort under fatigue.

## 2 Methods

### 2.1 Study design

This study was part of a larger project that compared two prototype shoes (66). Beyond the footwear comparison, we sought to explore the psychophysiological responses occurring during trail running in an exploratory manner. More information regarding the comparison of footwear conditions can be found elsewhere (29).

A randomized and counterbalanced design was used in the larger project, with each participant completing two testing sessions (one per shoe condition) separated by between three and 11 days. For the purposes of the current study, and to keep the data as rich as possible, both shoe conditions were kept, and our analysis controlled for shoe type, and the repeated nature of the data. Individuals expressing an interest in the study completed a screening questionnaire to confirm eligibility (see Section 2.2). Those eligible to participate received standardized pre-test instructions: avoid strenuous activity, alcohol, and caffeine in the 24 h preceding the test; arrive rested, hydrated, and having eaten their last meal at least two hours before. Participants were also asked to maintain consistent nutrition and training load across sessions. The study was approved by the Human Research Ethics Committee from University of Canberra (No. 202413875) and conducted in accordance with the Declaration of Helsinki.

### 2.2 Participants

Fourteen experienced male trail-runners volunteered to participate (age 25.3 ± 5.7 years; height 179.3 ± 5.0 cm; mass 70.7 ± 6.5 kg). Based on McKay et al. (30), participants were classified as trained to highly trained (Tier 2–3), with a mean weekly training volume of 13.9 ± 5.6 h and an International Trail Running Association (ITRA) performance index of 738 ± 90. ITRA data were unavailable for two participants. All participants met the inclusion criteria: ≥18 years old, comfortable running at a 16 km.h^−1^ pace on flat terrain, accustomed to running >2 h in duration, training >40 km per week, no musculoskeletal injury impacting movement, and shoe size EU 44.

### 2.3 Experimental procedure

After providing informed consent, participants completed questionnaires on demographic information, training load, and motivation to perform [Motivation State Questionnaire; (27)]. Participants were then randomly assigned to one shoe condition for the first session and wore the alternate pair in the second session.

**Figure 1:**
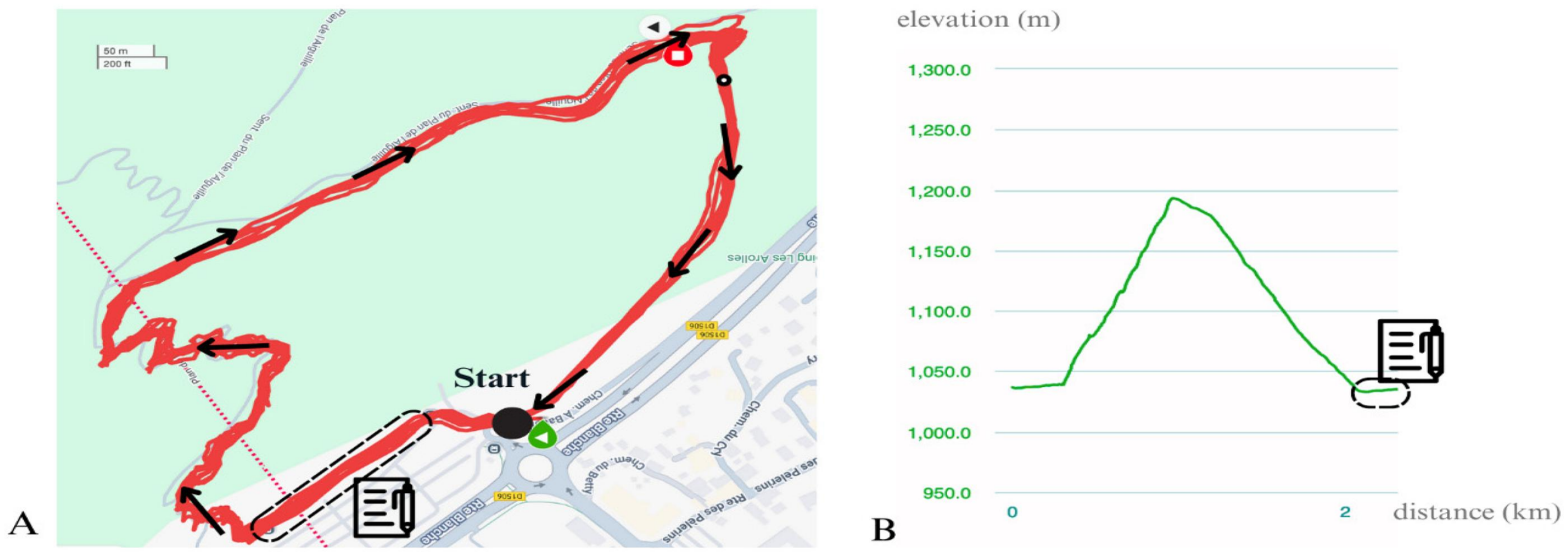
Running task course map **(A)** and gradient **(B)** with timing of collected perceptual measures (dotted lines).

Before testing, athletes were weighed and fitted with a heart rate monitor. The warm-up consisted of a lap of the 2 km test course, guided by a researcher to ensure familiarity. Each participant then completed a 90-minute run at a target perceived exertion of 14 on Borg's 6–20 scale (31), consistent with the average exertion reported during trail races (32). The course included uphill (+24%), downhill (−14%), and flat sections (Figure 1). On each lap's flat section, perceptual measures were collected in the following order: RPE, RPE-leg, affective valence, arousal, and motivation with the corresponding scales presented to participants. Responses were recorded via microphone by the same researcher throughout, to minimize bias. Water was provided concurrently. All participants were familiarized with the scales before the test. To ensure a total running time close to 90 min, an alternative shorter route was used if insufficient time remained to complete a full loop.

### 2.4 Measurements

#### 2.4.1 Pre task motivation

Motivation was assessed using a 14-item Motivation Scale (27) which conceptualises motivation as a task-related psychological state reflecting an individual's engagement and willingness to invest effort during performance. It comprises two distinct subscales: success motivation, reflecting the desire to achieve high performance or succeed, and interest motivation, reflecting intrinsic enjoyment and interest in the task itself. Each item was rated on a 5-point Likert scale ranging from 0 (*not at all*) to 4 (*extremely*). Subscale scores range from 0 to 28, with higher scores indicating stronger motivation. This measure was administered prior to task execution to assess participants' baseline motivational state and to ensure they were sufficiently motivated to engage with the task. In line with Motivation Intensity Theory (4), this step was important to confirm that participants were willing to invest effort, allowing subsequent effort-related responses to be interpreted as meaningful task engagement rather than disengagement or lack of motivation. In this sense, the pre-task measure reflects participants' potential motivation, defined as the maximum amount of effort an individual is willing to exert to satisfy a motive (3).

#### 2.4.2 In-task perceptual metrics

##### In task motivation

In-task motivation was assessed using a single-item measure based on Wright's (3) concept of potential motivation. Whereas the pre-task measure captures baseline motivational orientation, this item was designed to capture moment-to-moment fluctuations in motivational engagement during task performance. Participants rated their willingness to exert effort on a 5-point Likert scale in response to the question: “How willing are you to exert effort?” (1 = “not at all”; 5 = “extremely”).

##### Rating of perceived exertion (RPE)

Perceived exertion was assessed using Borg's 6–20 scale (31), with participants rating “how hard, heavy, and strenuous the physical task currently is”. This measure was used to verify compliance with the clamped protocol. During the first loop, if athletes deviated substantially (<10 or >15) from the target intensity (RPE = 14), they were reminded to adjust their effort accordingly.

##### Leg RPE (RPE-leg)

Leg RPE was assessed using Borg's CR10 scale (33), where participants rated “How hard is it for you to drive your leg” on an 11-point scale (0 = “no exertion at all”; 10 = “extremely strong”). This measure was initially included to compare shoe performance but later discussed in relation to change in affect related to body sensations.

##### Affective responses

Affective responses were assessed through measures of valence and arousal. Affective valence was measured with the Feeling Scale [FS; (34)], a single 11-point bipolar item ranging from −5 (“very bad”) to 5 (“very good”) anchored at −3 (“bad”), −1 (“fairly bad”), 0 (“neutral”), 1 (“fairly good”), and 3 (“good”). Valence was explained as a feeling of pleasure/displeasure experienced while running. Arousal was measured with the Felt Arousal Scale [FAS; (35)], a 7-point scale (0 = “low arousal”; 6 = “high arousal”). Arousal was explained as a state of activation with high arousal being in a highly activated state such as after drinking five cups of coffee and low arousal associated with feelings of relaxation or calmness.

#### 2.4.3 Physiological workload: data collection and processing

Physiological workload was estimated by calculating the HR:Speed ratio as an index of dissociation between internal workload (heart rate) and external workload (running speed). Heart rate and running speed were recorded using a GPS Watch (Garmin Forerunner 945. Garmin Ltd) at 1 Hz. Data were exported and linearly interpolated to impute short missing segments (1–8 s, ∼50% per file on average). Visual inspection of GPS and heart rate traces confirmed interpolations produced plausible values. Of the 28 participant-sessions (14 × 2), one session file was removed due to noisy heart rate data, and three laps from another participant were excluded due to implausible values. The final dataset included 150 full loop data points and 123 flat section data points. For each loop, HR:Speed ratio was calculated as mean heart rate (bpm) divided by mean speed (km·h^−1^) on the full loop data, and this process was repeated for the flat section data.

### 2.5 Statistical analysis

All analyses were conducted using R (version 4.5.1; RStudio 2025.05.1+513, Posit Software). Models were fitted using a Bayesian framework via the *brms* package (36) with non-informative flat priors. Non-informative priors were specified for fixed effects to allow posterior estimates to be driven primarily by the observed data, consistent with the exploratory nature of the present analyses.

Threshold (intercept) parameters and group-level standard deviations were assigned weakly informative Student-t priors with 3 degrees of freedom, mean 0, and scale 2.5. However, we note that in small samples and ordinal models, such priors can lead to unstable or extreme estimates with high uncertainty and therefore results should be interpreted with caution. We believe this limitation is more likely to affect the precision with which the magnitude of the effects can be quantified, rather than the overall direction of the observed relationships. An *a priori* sample size calculation was not performed, as the present analyses were conducted on an existing dataset originally collected for a separate study with a different primary objective. As a result, sample size was determined by the original study design and practical constraints inherent to prolonged, field-based endurance testing. Within the Bayesian framework adopted here, inference does not rely on null-hypothesis significance testing or statistical power considerations, but rather on the estimation of posterior distributions and the explicit quantification of uncertainty. Accordingly, all results are interpreted in light of the associated uncertainty rather than binary significance thresholds. Convergence diagnostics are provided in Supplementary File.

#### 2.5.1 Physiological workload

Generalised additive mixed models assessed changes in heart rate, speed, and HR:Speed ratio and RPE across % task completion. Response variables were modelled as smooth functions using thin-plate regression splines with up to 5 basis functions. The HR:Speed slope was approximately linear, and is reported as posterior mean ±95% equal-tailed credible interval. Full-loop HR:Speed ratios were used to characterize physiological workload, while flat-section ratios were used for mediation analyses involving psychological measures for better alignment and more controlled context.

#### 2.5.2 Perceptual measures

Cumulative link mixed models were applied to ordinal outcomes (affective valence, arousal, motivation) across task progression. Effects are reported as posterior mean log-odds and odds ratios (OR = e^log-odds^), representing the multiplicative change in odds of reporting a lower ordinal category per 1% task progression.

#### 2.5.3 Mediation analysis

**Figure 2:**
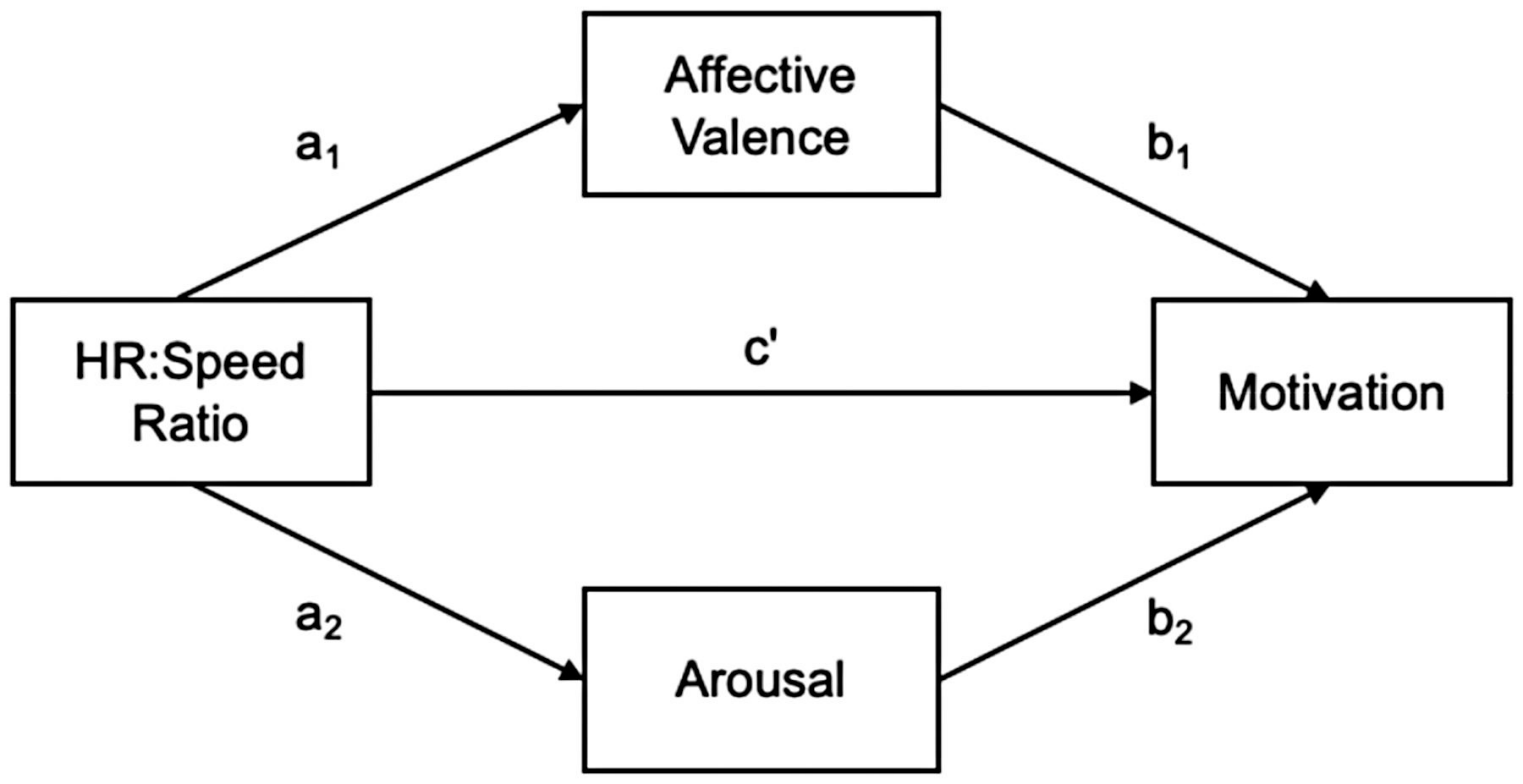
Parallel mediation model specification.

A parallel mediation model (37, 38) was used to examine potential indirect effects of HR:Speed ratio on motivation via affective valence (a_1_) and arousal (a_2_), while also estimating the direct effect (c′) (Figure 2). As both mediators and outcome were ordinal, a cumulative link model was used. HR:Speed ratio was mean-centred for modelling. Effects for paths a_1_ and a_2_ are presented as the posterior mean change in log-odds and odds ratios for a 1-unit change HR:Speed ratio. Mediation effects are described in Table 2.

#### 2.5.4 Model structure

Participant and session were included as random factors in all models to account for the nested data structure. As footwear has been shown to influence perceptual responses (29), footwear was also included as a random factor. Posterior distributions are summarized with 95% credible intervals and the probability of direction (PD), representing the probability that an effect is positive or negative.

## 3 Results

### 3.1 Session characteristics

Participants completed an average of 6 ± 1 full laps, covering 13.24 ± 1.48 km and 1,008 ± 133 m of elevation gain per session. Individual lap counts ranged from 5 to 7, with distances between 10.49 and 16.33 km, and elevation gains from 802 to 1,297 m. Environmental conditions were 24.4 ± 3.8 °C and 66.5 ± 14.8% humidity. Baseline motivation scores indicated a highly motivated sample (success motivation: 20.5 ± 4.1; intrinsic motivation: 24.0 ± 3.3). Descriptive characteristics for the first and last laps are presented in Table 1.

**Table 1:** Descriptive statistics of mean values ± SD for 1st lap and last lap for each variable.

Metrics	1st lap	Last lap
Heart rate (bpm)	158 ± 9	163 ± 11
Speed (km.h^−1^)	8.77 ± 0.94	8.60 ± 1.11
HR:Speed ratio	18.18 ± 2.25	19.31 ± 2.79
RPE (6–20)	12.6 ± 1.4	14.7 ± 1.0
RPE-leg (1–10)	3.5 ± 2.3	6.0 ± 2.1
Valence (−5 to 5)	3.2 ± 0.9	1.7 ± 2.2
Arousal (1–6)	3.9 ± 1.2	3.9 ± 1.1
Motivation (1–5)	4.4 ± 0.6	4.0 ± 1.1

**Figure 3:**
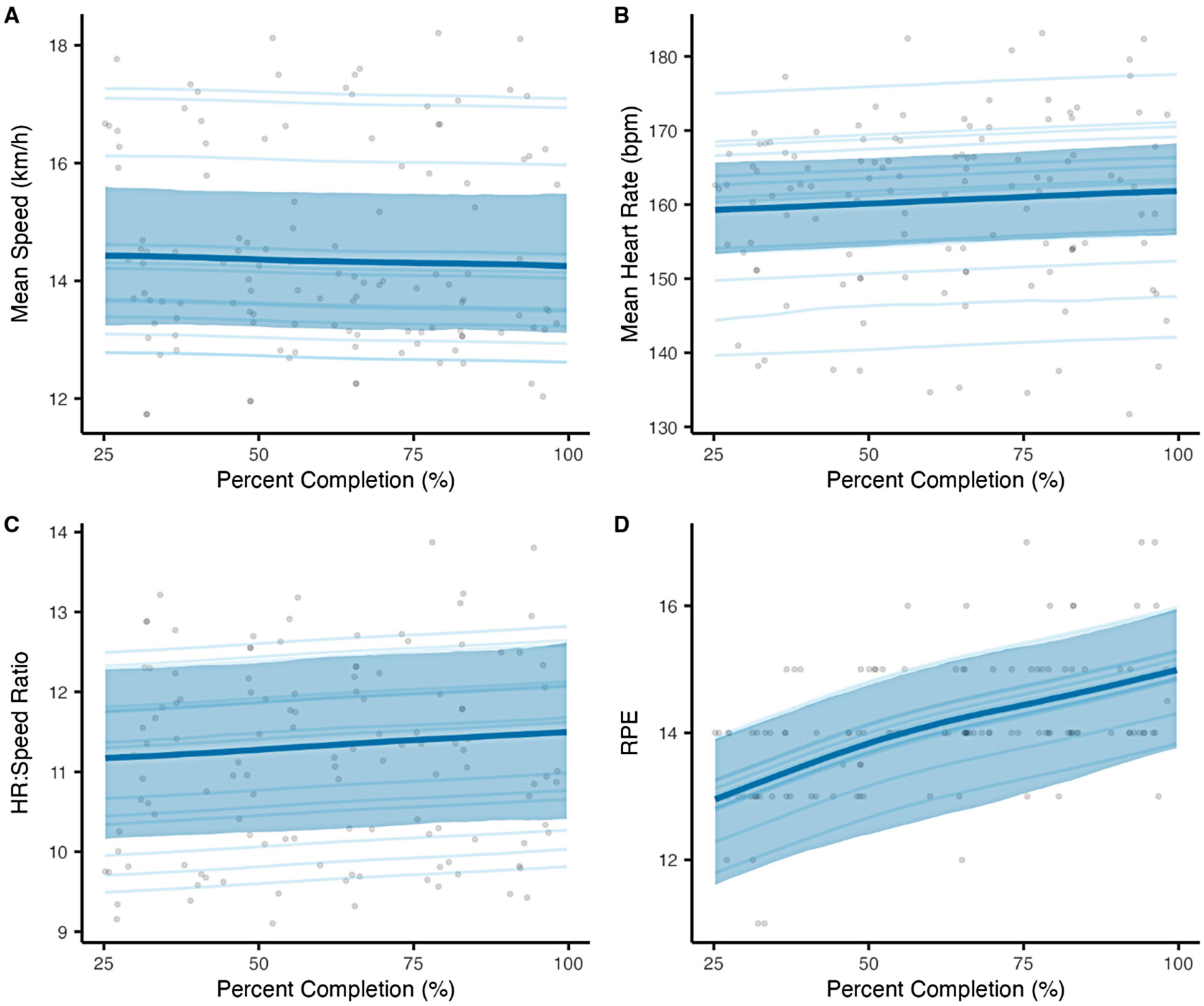
Physiological workload across flat sections. **(A)** average speed, **(B)** average heart rate, **(C)** HR:Speed ratio and **(D)** RPE. Grey points show observed values. Thick dark blue line represents the estimated population-level mean with 95% credible intervals (shaded ribbon). The thin light blue lines represent the model predicted trajectories for each participant in each session.

The HR:Speed ratio increased linearly across the running task [slope = 0.02 (0.01, 0.021), PD = 1.00] corresponding to an increase of 2 units in the ratio by task completion. In the flat section, the increase was smaller [slope = 0.004 (0.001, 0.007), PD = 0.99] (Figure 3) equivalent to a 0.4-unit rise. The average RPE was 13.8 ± 0.8 with a linearly increase over time [slope = 0.026 (0.020, 0.032), PD = 1.00], corresponding to a 2.6-unit rise by the end of the prolonged run. Affective valence declined progressively, with 5% higher odds of reporting a lower valence score than higher valence score for every 1% task progression [log-odds = 0.05 [0.03, 0.06], OR = 1.04 [1.03, 1.06] PD = 1.00]. Arousal remained stable [log-odds = 0.00 [−0.01, 0.01], OR = 1.00 [0.98, 1.01], PD = 0.53]. Motivation also declined, with a 3% higher odds of reporting a lower motivation score (than higher motivation score) every 1% progression through the task [log-odds = 0.03 [0.04, 0.01], OR = 1.03 [1.00, 1.04], PD = 0.99].

### 3.2 Physiological workload, affects and motivation

**Figure 4:**
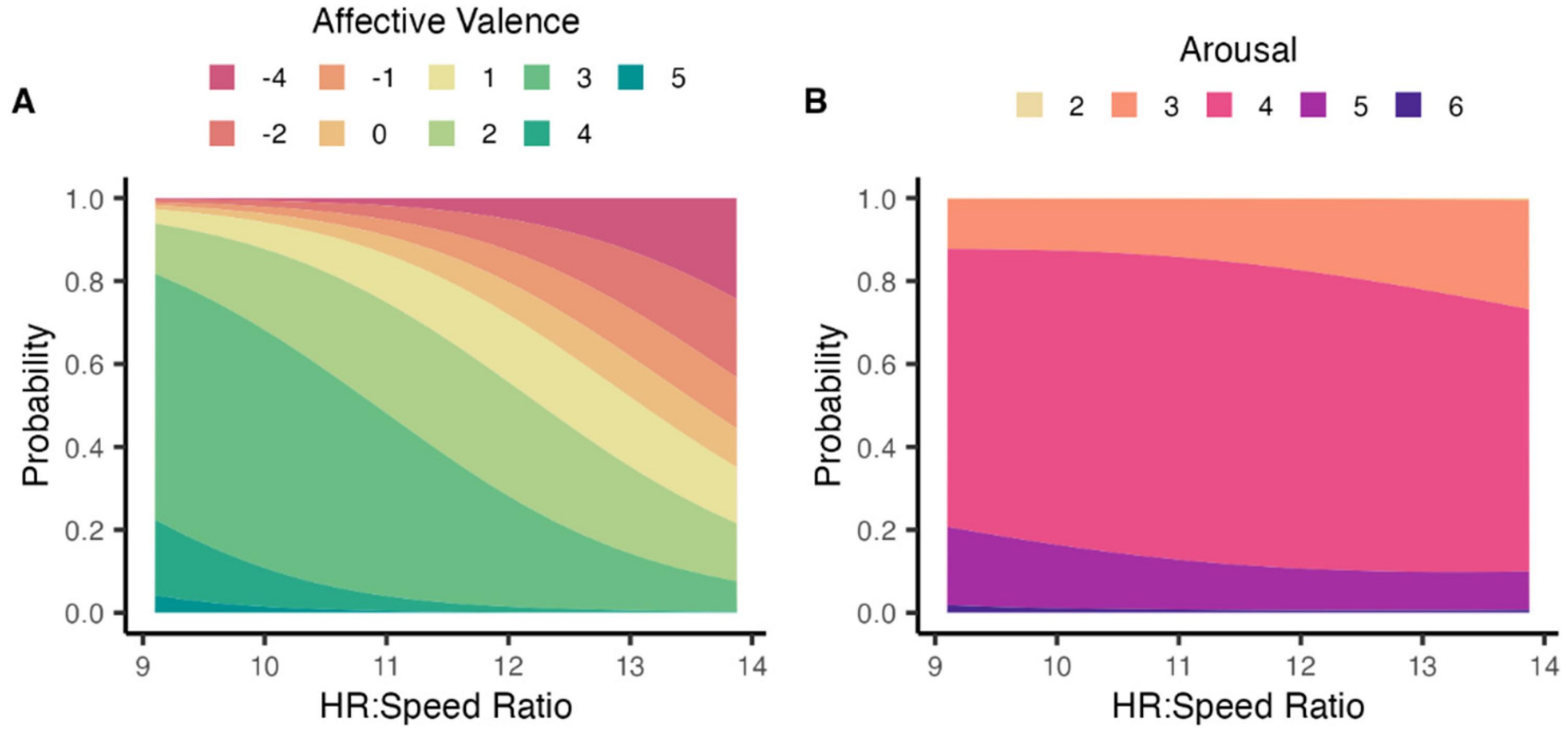
Predicted probabilities of affective valence (**A**), and arousal (**B**) as a function of HR:Speed ratio, corresponding to paths a_1_ (HR:Speed ratio → valence) and a_2_ (HR:Speed ratio → arousal) in the mediation model.

Path a_1_ and a_2_, representing the association between physiological workload and affective responses showed that higher HR:Speed ratios were strongly associated with lower valence [log-odds = −1.16 [−1.89, −0.49], OR = 0.31 [0.15, 0.62], PD = 1.00]. For each unit increase in HR:Speed ratio, the odds of reporting a lower valence category increase by 3.2 times. In contrast, evidence for association between arousal and physiological workload was weak [log-odds = −0.27 [−1.34, 0.71], OR = 0.76 [0.26, 2.04], PD = 0.70] (Figure 4).

**Figure 5:**
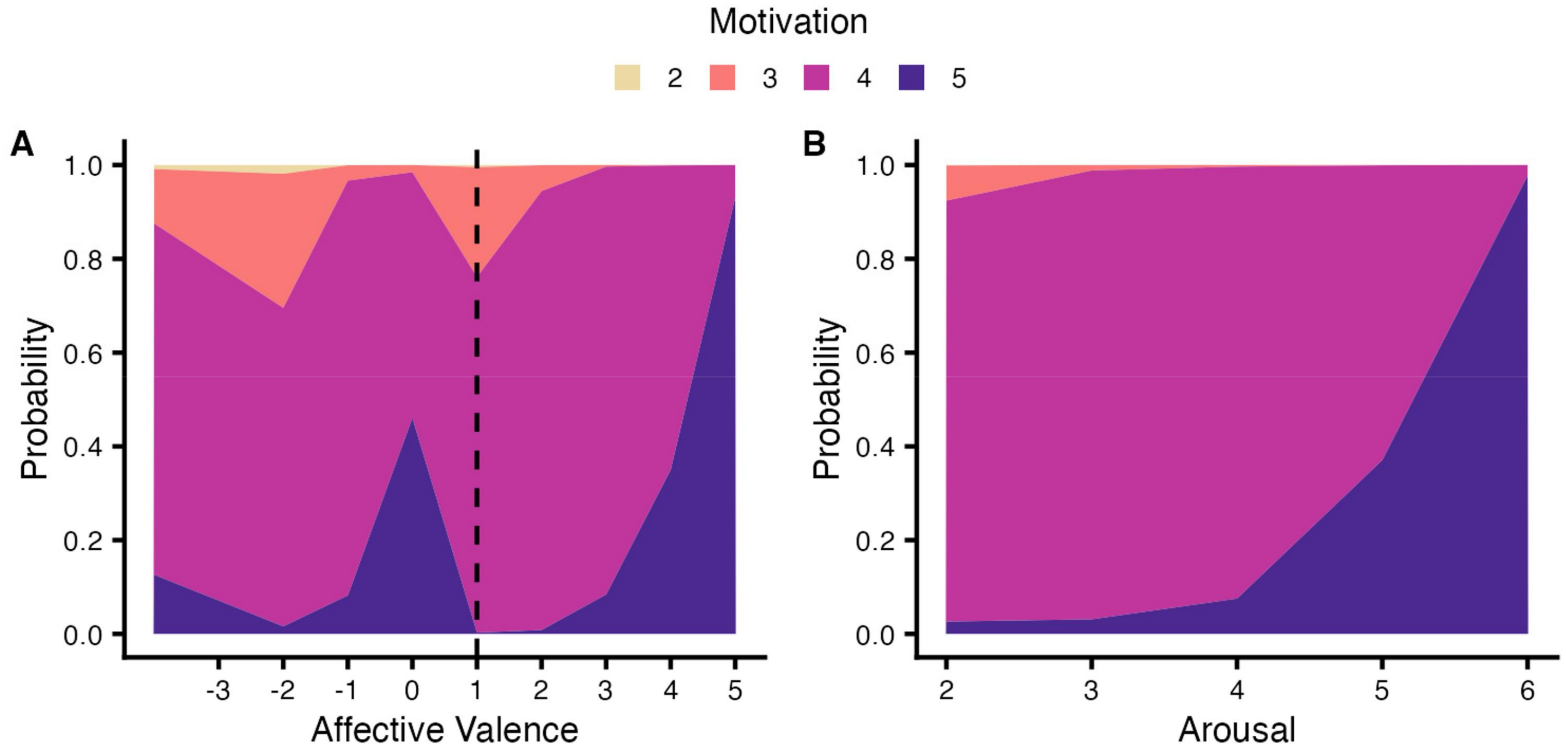
Predicted probabilities of motivation as a function of affective valence **(A)** and arousal **(B)**, corresponding to paths b_1_ (valence → motivation) and b_2_ (arousal → motivation) in the mediation model. Estimates are shown with HR:Speed ratio held at its mean value, while the alternate mediator was fixed at its typical distribution at the mean HR:Speed ratio.

Paths b_1_ and b_2_, representing the association between affective responses and motivation, indicated that valence and arousal tended to be positively associated with motivation. Higher valence [log-odds = 18.53 (1.03, 49.35), PD = 0.99] and higher arousal [log-odds = 11.17 (4.14, 21.51), PD = 1.00] were generally associated with higher motivation scores. However the wide credible intervals and inflated estimates indicate substantial uncertainty regarding the magnitude of these associations, likely due to sparse cell distributions and evidence of quasi-separation in the data. Predicted probabilities should therefore be interpreted cautiously in sparsely populated regions. Figure 5 provides a more interpretable representation of the general direction and pattern of the associations than the log-odds parameters alone. This includes rare affective valence values below 1 (see area below the dotted line in Figure 5) and rare occurrences of motivation values below 3. Predictions in this area are consequently less stable. In contrast, motivation was consistently observed at its highest level (5) when affective valence reached its maximum (5), and was predominantly observed at its highest level when arousal was at its maximum (6), resulting in high predicted probabilities in these regions. As such, these findings should be interpreted as evidence of a probable positive relationship rather than precise estimates of effect size.

**Figure 6:**
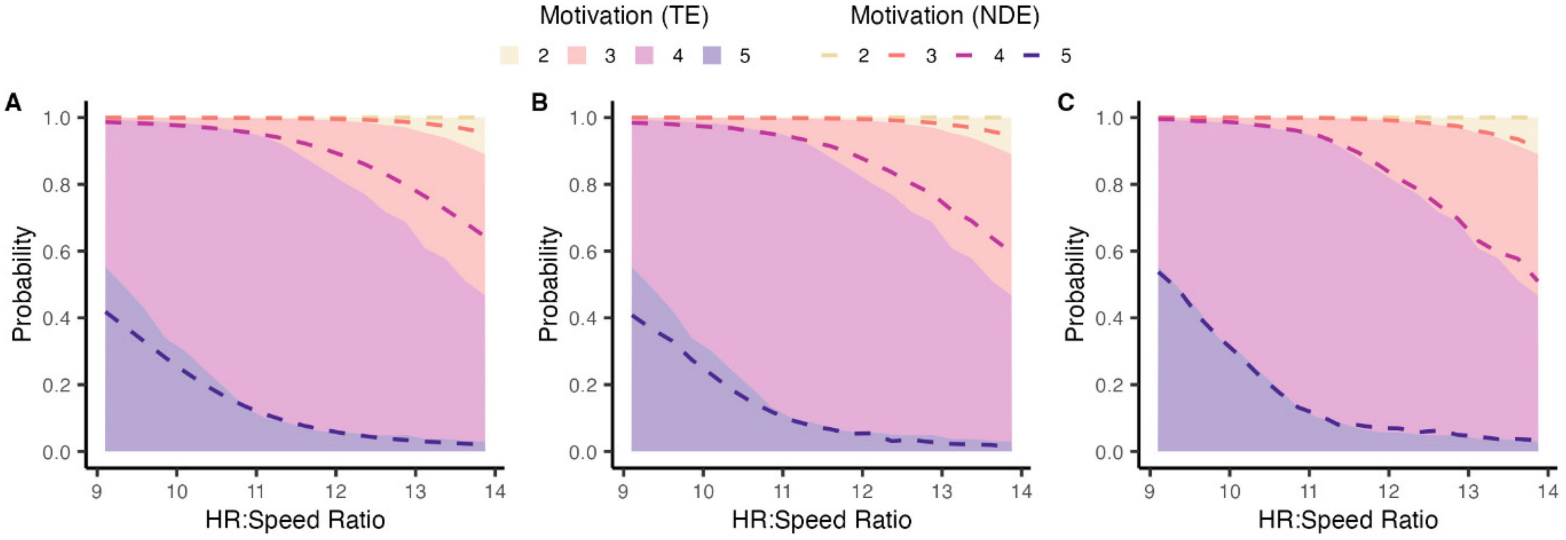
Path c. Predicted probabilities of motivation category as a function of HR:Speed ratio, illustrating the Total Effect (TE; affect allowed to vary naturally; as solid lines) and Natural Direct Effect (NDE; affect fixed at typical levels; as dotted line). The difference between the TE and NDE represents the Natural Indirect Effect of the mediator. **(A)** NDE estimated with both mediators (valence and arousal) are held at their typical distribution at the mean HR:Speed Ratio. **(B)** NDE estimated with valence fixed and arousal allowed to vary naturally. **(C)** NDE estimated with arousal fixed and valence allowed to vary naturally.

Path c, representing the effect of physiological workload on motivation, indicated that a + 1 increase from the mean HR:Speed ratio was associated with a total effect (TE) of −0.19 (i.e., a 0.19-point decrease in motivation), comprised of a natural direct effect (NDE) of −0.13, and a total indirect effect (NIE) of −0.06. This suggests that 32% of the total effect was attributable to affective responses, with affective valence accounting for 28% of the total effect (NIEvalence = −0.05) while arousal accounted for 4% (NIEarousal = −0.01). Mediation effects with a comparator of +1 in the HR:Speed ratio are reported in Table 2. However, since the change from the mean was not strictly linear, this comparator does not represent the dynamic nature of this effect and should be interpreted in combination with Figure 6, which provides a visual representation of these dynamics. Inspecting Figure 6, the probability of reporting lower motivation increased with higher HR:Speed ratios. At lower HR:Speed ratios, valence was naturally high, and allowing it to vary accordingly resulted in higher motivation compared to when valence was fixed at its mean value for that HR:Speed level. This is reflected by the Total Effect curve lying above the Natural Direct Effect curve, indicating that naturally elevated valence at low HR:Speed ratios facilitated motivation. Conversely, at higher HR:Speed ratios, declining valence reduced the likelihood of reporting high motivation, as shown by the Total Effect curve falling below the Natural Direct Effect curve (Figure 6A). Patterns were nearly identical when arousal was held constant (Figure 6C), suggesting that valence accounted for most of the indirect effect.

**Table 2:** Mediation effects of a + 1 increase in HR:Speed ratio relative to the mean (11.1 ± 1.2).

Effect	Description	Mean	95% CI	PD
Total effect (TE)	Overall effect of HR:Speed ratio on motivation, with affective valence and arousal allowed to vary naturally.	−0.19	−0.46, 0.00	0.97
Natural Direct Effect (NDE)	Isolated effect of HR:Speed ratio on motivation when affective valence and arousal are held fixed at the typical distribution at the mean HR:Speed ratio.	−0.13	−0.35, −0.01	0.98
Total Natural Indirect Effect (NIE_total_)	Portion of the effect transmitted through affective valence and/or arousal.	−0.06	−0.23, 0.09	0.83
Natural Indirect Effect via affective valence (NIE_valence_)	Portion transmitted exclusively through valence, with arousal fixed	−0.05	−0.21, 0.10	0.82
Natural Indirect Effect via arousal (NIE_arousal_)	Portion transmitted exclusively through arousal, with valence fixed	−0.01	−0.07, 0.03	0.68
Indirect Interaction Portion	Portion arising from the joint, non-additive action of valence and arousal. [NIEtotal—(NIEvalence + NIEarousal)]	0.00	−0.01, 0.01	0.59
Proportion mediated	Proportion of TE mediated by valence and arousal combined. (NIE_total_/TE)	0.32		
Proportion via affective valence	Proportion of TE mediated by valence. (NIE_valence_/TE)	0.28		
Proportion via arousal	Proportion of TE mediated by arousal. (NIE_arousal_/TE)	0.04		

Effects (TE, NDE, NIE) are expressed as the change in motivation units.

## 4 Discussion

The first aim of this exploratory study was to examine how physiological workload relates to changes in motivation during a prolonged trail run. Our results suggested that physiological workload related to speed may predict willingness to exert effort, with higher HR:Speed ratio generally associated with lower motivation (path c). These findings extend the limited existing work on in-task motivation fluctuations in endurance exercise. While most research has focused on pre-task motivation or long-term persistence and adherence, Baron et al. (6), demonstrated that the quality of motivation changes during incremental exercise. Specifically, as the task became more demanding, intrinsic motivation decreased substantially (−34.6%) while integrated regulation increased (+17.3%). This shift reflects a progressive transition from more self-determined to more controlled forms of motivation. Similarly, Taylor et al. (5) reported that the desire to reduce effort increased with rising exercise intensity. Although recent work provided initial evidence for a mediating role of affective valence (26), these findings were obtained under laboratory conditions and did not account for the broader affective experience, notably the potential role of arousal. To better understand the mechanisms underlying motivational changes during prolonged endurance exercise, we therefore explored whether affective responses, considering both valence and arousal, may contribute to the relationship between physiological workload and motivation. Given the exploratory nature of these analyses, the following sections discuss the potential respective contributions of affective valence and arousal to effort regulation.

### 4.1 Affective valence (path a_1_, path b_1_ and mediation role)

In the present study, the progressive increase in the HR:Speed ratio throughout the task captured the dissociation between internal and external workload, indicating a rising physiological workload (9, 11). This dissociation was characterized by an increase in internal workload, reflected by higher heart rate, alongside a concurrent decrease in external workload (running speed). As workload increased, affective valence tended to decrease (path a_1)_. This decline in valence is consistent with previous findings showing that prolonged exertion is typically accompanied by reduced pleasure (23, 39). The association between physiological workload and affective valence is well established, with pleasure declining as intensity increases, particularly near the ventilatory or metabolic threshold (21, 40), a range that aligns with the intended task intensity (RPE 14). Notably, the increase in RPE_leg suggests that sensations of leg heaviness may have acted as an aversive interoceptive signal, impacting affective valence. Additionally, given the competitive profile of our participants, reflected in their high scores for success motivation, the decline in valence may also reflect a negative psychological response to reductions in running speed, and a conscious awareness of and dissatisfaction with not sustaining their intended performance level (41).

Affective valence tended to impact motivation (path b_1_), with higher pleasure associated with higher motivation. However, credible intervals were large, indicating substantial uncertainty and warranting cautious interpretation regarding the magnitude of this effect. Consistent with these patterns, the mediation model suggested that valence may play a prominent role in the relationship between physiological workload and motivation, as indicated by its mediating contribution (28% of the total effect). When physiological disturbance is low, positive valence appears to support motivation. In the contrary, as physiological disturbance increases, running becomes progressively more aversive, leading to declines in valence that contribute to lower willingness to exert effort. From an approach–avoidance perspective, increasing physiological disturbance is likely perceived as increasingly less pleasant, eliciting a motivational tendency to avoid or disengage from the aversive stimulus (42, 43).

### 4.2 Arousal (path a_2_, path b_2_ and mediation role)

Physiological workload appeared to have little impact on arousal, which remained stable despite increases in the HR:Speed ratio (path a_2_). To date, relatively little research has examined arousal in relation to physiological workload relative to speed, as most studies have instead investigated arousal in controlled laboratory settings using fixed workloads. Accordingly, we described the relationships between arousal, heart rate, and running speed separately.

The observation that arousal remained stable despite increases in heart rate is noteworthy, as arousal as traditionally been indexed through cardiovascular responses (44), implying that rising heart rate should correspond to higher perceived arousal. However, recent work has challenged the notion of arousal as a unitary physiological state, showing that it cannot be inferred from a single physiological marker such as heart rate (45). The association between heart rate and arousal may therefore hold at the onset of exercise, when athletes mobilise resources and both increase; a pattern observed by Van Landuyt et al. (46). Supporting this interpretation, we previously reported that arousal may reflect heightened attentional resource allocation rather than increased physiological load, with higher arousal observed during downhill running compared with flat or uphill gradients despite lower heart rate responses (29). Once the task is underway, however, theorical frameworks such as the Inverted-U (47) and Catastrophe theory (48), suggest that athletes operate within a functional arousal zone that supports stable performance.

When considering changes in running speed, arousal remained stable while performance declined. Under competitive circumstances, one might expect athletes to allocate additional resources, and thus increase arousal, to maintain performance. However, participants were instructed to maintain an RPE of 14. Because they had no incentive to protect speed and were running at an intensity below their glycolytic threshold where homeostasis disturbance is relatively controlled, they likely did not need to increase arousal to remain at the prescribed intensity. The stable arousal observed here may therefore reflect effective regulation within this functional range, even as physiological workload increased. This suggest that in prolonged exercise performed under RPE-pacing, perceived arousal may be driven more by task demands, attentional resource allocation, and deliberate effort regulation than by physiological strain itself.

Arousal seemed to have a minimal contribution to the relationship between physiological workload and motivation (4% of the total effect). One likely explanation is that physiological workload did not alter arousal (path a_2_). If arousal remains stable, it cannot transmit the effect of physiological workload onto motivation, which accounts for its negligible role in the mediation model. Additionally, literature provides no evidence for a direct causal link between arousal and motivation (49). Reversal Theory nonetheless offers a useful interpretative lens, suggesting that motivational state shapes how arousal is experienced. When the needs for a given motivational state are not met, reversals may occur, leading the same physiological arousal to be interpreted as pleasant or unpleasant depending on motivational orientation. Kerr and Vlaswinkel (50) for instance, observed that faster runners often began in a goal-oriented state but shifted into a playful state mid-run. These findings may illustrate that motivational states fluctuate dynamically during endurance exercise, altering the emotional meaning of physiological activation without requiring changes in arousal itself. Within this context, and under the presented RPE-clamped protocol, arousal does not appear to be a critical regulatory variable. Instead, its role seems secondary to the interplay between physiological disturbance, affective valence, and motivational orientation.

### 4.3 Practical implication and future directions

Previous literature grounded in motivational intensity theory suggest that maximal effort investment depends on potential motivation. In contrast, the present study provides preliminary evidence that during prolonged exercise, physiological workload can itself decrease motivation, an effect strongly mediated by affective valence. As physiological disturbances become increasingly aversive, motivation tend to decline, increasing the potential likelihood of task withdrawal.

From a practical perspective, these findings highlight the importance of strategies aimed at stabilising or enhancing affective experience during prolonged exercise. Even independently of physiological workload, positive affect can offset perceived exertion and support sustained performance (51), with perceived pleasure explaining up to 30% of pacing variability in trail running (17). In competitive settings, affective responses may be further challenged by factors unrelated to workload, such as pain, gastrointestinal discomfort, nutritional errors, environmental stressors, or adverse weather conditions, while arousal may fluctuate due to terrain, monotony, or sleep deprivation. If translatable outside the clamped-RPE protocol, our findings would suggest that when these factors deteriorate affective experience, they may undermine willingness to continue, thereby increasing the importance of interventions targeting affect regulation.

Promising strategies include improving interoceptive accuracy (52), including subliminal priming (51), leveraging environmental or social cues [e.g., running with an opponent (53);], mindfulness-based approaches (54) and ergogenic aids such as caffeine (55). Emerging evidence also suggests that prior exposure to discomfort can recalibrate affective responses during exercise (56). Contextual factors such as pre-task core-affect (57, 58) and footwear characteristics (29) should also be considered when designing interventions aimed at optimizing motivation and performance.

Future work should explore whether individual differences moderate the relationship between affect, physiological workload, and motivation, with a focus on factors that may be relevant in applied settings. While personality traits do not appear to strongly influence affective responses to strenuous exercise (59), characteristics such as resilience, emotional intelligence (60), motivational profile, intensity tolerance and preference (64) and pre-exercise psychological state may shape how athletes regulate affect and sustain motivation under fatigue. Together, this line of research may help inform interventions aimed at preserving affective valence and endurance performance, by supporting pacing decisions and helping guide support crews in identifying and addressing early signs of motivational decline.

### 4.4 Limitations

This study presents several limitations that should be acknowledged. Firstly, the use of a field-based RPE-clamped protocol introduces methodological considerations that differ from controlled laboratory settings. Although participants were instructed to maintain a constant perceived exertion, RPE increased progressively throughout the task. Rather than reflecting non-compliance, this pattern likely represents an adaptive pacing strategy in which athletes begin conservatively and gradually increase effort as they approach task termination, a process well documented in endurance performance (61, 62). It is also possible that changes in valence were partly driven by these shifts in RPE. Future work employing alternative protocols may help disentangle these relationships and provide deeper insight into the mechanisms linking perceived exertion and affective responses. Secondly, in-task perceptual ratings may have been influenced by external environmental and contextual factors, including encounters with other trail users (e.g., dogs and humans) and discomfort caused by the distal-tibia IMU worn for a separate study. While these factors may introduce additional variability, they also reflect the ecological complexity of real-world trail running, where affective and physiological responses are shaped by concurrent environmental influences. Third, the use of a single-item measure to assess in-task motivation, although informed by a more theoretically grounded and comprehensive baseline assessment using the Matthews (27) scale, may limit the generalisability and interpretability of the findings, as it does not capture the multidimensional nature of motivation. Future work should therefore extend more robust multi-item approaches to in-task measurement to better align state motivation assessment with its theoretical complexity. Finally, the relatively small sample size and the inclusion of only trained male participants (due to footwear size constraints), limit the generalisability of these findings. Accordingly, caution is warranted when extrapolating these results to less trained populations. Future work should seek to replicate these findings in larger samples.

## 5 Conclusion

The findings of this exploratory study suggest that physiological workload relative to speed may influence willingness to exert effort partly through its association with affective responses. Affective valence appears to play an important role, potentially reflecting whether the rising cost of effort should be approached or avoided. However, given the uncertainly and instability of the models, the findings should be interpreted with caution. Despite these limitations, the results highlight the potential relevance of monitoring both physiological and affective indictors during a race, as they may help identify early signs of declining motivation that could impair performance. Such information could be used by athletes to better regulate their effort, and by support crews to adjust strategies and provide timely interventions.

## Data Availability

The raw data supporting the conclusions of this article will be made available by the authors, without undue reservation.
