# Supplementary material for: From physiological workload to motivation during a prolonged trail run: the mediating role of affective valence and arousal
Source: Front Sports Act Living. 2026 Jul 1;8:1805336. doi: 10.3389/fspor.2026.1805336 (PMC13370938; doi:10.3389/fspor.2026.1805336)
Supplement: Supplementary file 1 [file Datasheet1.pdf]

Convergence diagnostic:

Diagnostics indicated acceptable convergence, with all R-hat values at 1.0 or 1.01 (for some motivation terms). Effective sample sizes (ESS) were also acceptable, with bulk ESS ranging from 921 – 2314 and tail-ESS ranging from 809 – 2806 (given that four chains were used, a minimum target ESS of ~ 400 has been recommended; (Vehtari et al., 2019). Slightly lower ESS values (~800-1000) were observed for some motivation-related terms, but these remained above recommended minimum thresholds.

Reference: *Aki Vehtari, Andrew Gelman, Daniel Simpson, Bob Carpenter, and Paul-Christian Burkner (2019). Rank-normalization, folding, and localization: An improved R-hat for assessing convergence of MCMC. arXiv preprint arXiv:1903.08008.*
